# Economic development through women’s economic rights: a panel data analysis

**DOI:** 10.1007/s10368-023-00560-1

**Published:** 2023-04-18

**Authors:** Amjad Naveed, Nisar Ahmad, Amber Naz, Aziza Zhuparova

**Affiliations:** 1 Department of Business Development and Technology, Aarhus University, Herning, Denmark; 2 Department of Economics and Finance, Sultan Qaboos University, Seeb, Oman; 3 Al-Farabi Kazakh National University, Almaty, Kazakhstan

**Keywords:** Sustainable development, Women economic rights, Economic growth, Spatial econometrics

## Abstract

Increasing gender equality and enhancing women empowerment through work opportunities are the important steps in achieving sustainable development. The aim of this paper is threefold: (1) to empirically examine the relationship between women economic rights and economic growth for the global sample. (2) To explore whether the effect of women’s economic rights is different across different economies. (3) This paper uses spatial econometric techniques to examine the impact of women’s economic rights on neighbouring countries. For empirical purpose, we use the data for 171 countries over the period 1960–2016. The results show that women’s economic rights positively affect growth; however, the effect is heterogeneous across different economies. From spatial analysis, we confirm that 75% spillover effect of women economic rights is passing through neighbouring country. The results of this study are consistent and coherent with the EU policy about women’s economic empowerment where it claims that joint effort in promoting women empowerment by all actors could lead to sustainable development and growth.

## Introduction

Since the last decade, the concept of sustainable development has gained enormous attention among researchers and policymakers. Particularly, sustainable development goals (SDGs) announced by the United Nation (UN) have focused that gender equality and women’s rights are one of the important pillars of sustainable development (see SDG#5 in UN SDGs, 2015). Gender inequalities and fewer opportunities for women to work are still deeply rooted globally. Women do not have access to decent work and face inequalities in the form of both opportunities and wage gaps that might have adverse effect on economic growth of that country. Women’s empowerment implies that they have the same economic rights as men and are not discriminated against simply because they are female (Abigail and Moizza, 2017). Unfortunately, women are comparatively less privileged compared to men in terms of economic participation in society (Hassan and Cooray, 2015; Oztunc et al. 2015). Giving women economic rights can boost economic growth because it allows them to develop their potential as successful household managers without restriction (King & Mason, 2001; Sen, 1999). Women’s economic rights can bring long-lasting benefits across multiple dimensions. The UN Secretary-General’s High-Level Panel on Women’s Economic rights recently recognizes that:

“*Empowering women economically is not only the ‘right thing’ to do to honor the world’s commitments to human rights. It is also the ‘smart thing’ to do for development, economic growth and business*”.

(Klugman & Tyson, 2016)

Through various channels, such as trade and foreign direct investment, the degree of women’s economic rights in one country might occasionally influence the level of such rights in the neighbouring countries. Such effect is known as spatial dependence in the economic literature (Anselin, 1988; LeSage, 2008; Naveed and Ahmad, 2016). Concerning empirical literature, very few studies have tested spatial dependence related to women economic rights. For instance, Neumayer and De Soysa (2011) find a strong spillover effect of women economic rights for middle-income countries where the gender structure of employment in tradeable sectors is functioned by foreign investors. It implies that there are potential spillover effects from neighbouring countries through FDI, trade and other channels. Recently, Wang and Naveed (2021) explore the link between women empowerment and income inequality and find a strong relationship; however, they have not incorporated spatial analysis to capture the spillover effects from neighbourhood. Ignoring this can under or overestimate the true effect of women economic rights on economic growth (LeSage, 2008). The current study will thus particularly assess how the economic rights of women in neighbouring countries have an impact on economic growth.

Keeping in view the importance of women empowerment for growth, the main objectives of this paper are threefold. (1) To empirically examine the relationship between women economic rights and economic growth for the global sample. (2) To explore whether the effect of women’s economic rights is different across different economies. (3) This paper employs spatial econometric techniques to test the spatial dependence between the variable of interest. This is due to the fact that increasing women economic rights in one country might have spillover effect on its neighbouring countries due to the globalization, FDI and international trade (Elson, 1999; Neumayer & De Soysa, 2011; Wichterich, 2000). This interdependence among countries is formally known as spatial dependence in the literature which must be controlled for an appropriate analysis (Anselin 1988; LeSage 2008).

For empirical purpose, we use widely used estimation techniques, such as OLS, MLE and SDM (spatial Durbin model) to see the impact of women economic rights on economic growth. By using the panel data consists of 171 countries over the period 1960 to 2016, we find that women’s economic rights stimulates economic growth in the global sample. However, the effect is heterogeneous for the countries that differ in their income levels. Considering spatial model, we find that there is considerable spillover effect of women economic rights coming from neighbouring countries, i.e. 75% while the country’s own effect from women economic rights is only 25%.

This study offers critical factual proof, from a policy standpoint, that advancing women’s economic rights can support growth and pave the way for sustainable development. Particular focus should be paid to developing countries so that they can create greater employment options for women, especially in light of the recent pandemic (COVID-19). As a result, focusing on women’s empowerment through increased economic rights should be regarded as an appropriate growth strategy in the new millennium.

The remainder of the article is structured as follows. “Section 2” presents a review of literature related to women economic rights and economic growth. Methodology and empirical specification are explained in “Section 3”. “Section 4” provides a data description. Results are explained in “Section 5”, and the study is concluded in “Section 6”.

## Theory and literature

From theoretical perspective, many approaches have been developed in early 1970s and 1980s that are directly linked with women empowerment and economic development. For instance, Gender and Development (GAD) approach, Women in Development (WID) approach and rise of rights approach by NGOs (for detail, see Bradshaw et al., 2017). The WID approach highlights the importance of women and criticized why women are excluded from economic development process and why they have limited role in decision-making particularly for their education and employment, while the GAD approach argues that there should be balance in power for both men and women. Early 1990s, many non-governmental organizations (NGOs) emphasize the recognition that women’s demand is legitimate and they have rights to exercise their freedom without any restrictions. There are numerous theoretical and empirical studies that show how empowering women affects economic growth. For example, the most persuasive evidence on the role of women in economic development and growth comes from the World Bank’s research (see, Dollar and Gatti 1999; Klasen 2000). Following paragraphs report the review from empirical studies on women empowerment, women’s economic rights and its relation to economic growth.

According to Abigail and Moizza (2017), equal opportunities for gender have a substantial positive impact on capita income, economic development and national competitiveness. Woetzel (2015) suggests that gender equality could increase the global gross domestic product (GDP) by between $12 trillion and $28 trillion by 2025. Reduced gender disparities in employment and education also has a favourable impact on economic growth, which depends on labour market expansion and skill development. Domestic unpaid work mainly carried out by women is also vital to the social well-being and maintenance of the labour force. It is also noted that increasing women’s contribution to household income, through working, improves the wellbeing, reduces household poverty and reduces a family’s susceptibility to economic ups and downs. Few studies have shown that gender diversity in the board of a company reduces risk, increases productivity and has a positive effect on firm value (Campbell & Mínguez-Vera, 2008).

Oztunc et al. (2015) examined how women’s education affects long-term economic growth in the Asia-Pacific countries from 1990 to 2010. Using the panel regression analysis based on the theoretical and empirical literature, this study found that fertility rate, female labour force participation rate and female education had significant contributions in annual per capita income growth. On the empirical side, it also found that women’s education is a significant factor in economic growth.

Hassan and Cooray (2015) examined the long-run growth effects of education using a panel of eighteen Asian countries over the period 1970–2009. They also argued that female education had robust and relatively high growth effects by using both endogenous and exogenous growth frameworks. The study found that the average female enrolment ratios at the primary, secondary and tertiary levels are 83, 41 and 15% respectively as opposed to 98, 49 and 20% for males. Therefore, a significant gender gap also exists in male and female enrolment ratio in Asian countries.

Kabeer and Natali (2013) reviewed the impact of gender equality on economic growth and the impact of economic growth on gender equality. They argued that gender equality is an essential aspect of human dignity and social justice and is an effective means to achieve other development goals.

Rowland (2012) examined the influence of improved status on economic development for 126 countries every 5 years from 1980 to 2005. In addition, this study also developed the mechanism of the relationship of women’s status and economic development by using the pooled time-series cross-section analysis. This analysis incorporated several measures to assess the status of women such as education, labour force participation, fertility and infant mortality. The data is collected from the World Bank for all selected countries. They found that the effect of education and labour force participation on development was mediated by fertility and infant mortality.

According to Seguino (2000), GDP growth is positively related to wage inequality through its effect on investment. This study considers semi-industrialized export-oriented economies over the period 1975–1995. On the other hand, Fatima (2011) study utilized the OLS technique to show that there is no strong relationship between female education and GDP growth. The data is collected on GDP, investment, male education, female education and labour force participation from 1980 to 2006. Similarly, Cuberes and Teignier (2014) revealed that economic growth has a significantly positive impact on gender.

Klasen (2000) investigated to what extent gender inequality in education and employment may reduce growth and development by using cross-country and panel regression for 1960–1992. The result found that gender inequality in education has a direct impact on economic growth by lowering the average quality of human capital and an indirect impact on economic growth through investment and population growth.

In the empirical literature, very few studies have incorporated spatial econometric analysis. Table 1 shows the detailed contents of the paper we identified in the Scopus database related to economic development and women economics right. Based on our content analysis, we identified six empirical and three theoretical papers testing the relationship between economic growth and various types of women rights. The existing studies have mainly used logistic regression or various types of panel data model. As we notice, none of the papers has done spatial econometric analysis to capture the spillover effects from neighbourhood. This is very important to analyse how we gain or perceive from our neighbourhood and vice versa. Therefore, the current study will explore and quantify both direct effects (from country’s own women economic rights effect on growth) and indirect (spill-over effect from neighbouring countries).

**Table 1:** Overview of relevant studies based on women’s empowerment, economic development and growth (2000–2022)

Serial	Title	Type of paper	Country of analysis	Analysis time frame	Methodology	Source title	Authors	Year
1	Women’s political empowerment and economic growth	Empirical	182 countries	1831–2000	Panel fixed effects model	*World Development*	Dahlum S., Knutsen C.H., Mechkova V.	2022
2	Social and economic empowerment of women through financial inclusion: empirical evidence from India	Empirical	India	2018	Logistic regression	*Equality, Diversity and Inclusion*	Pal M., Gupta H., Joshi Y.C.	2021
3	Women’s empowerment and economic development: a feminist critique of storytelling practices in “randomista” economics	Theoretical	NA	NA	NA	*Feminist Economics*	Kabeer N.	2020
4	Do women’s advancement and gender parity promote economic growth? Evidence from 30 Asian countries	Empirical	30 Asian countries	1997–2015	Panel ARDL	*Millennial Asia*	Mishra P.K., Mishra S.K., Sarangi M.K.	2020
5	What influence do empowered women have? Land and the reality of women’s relative power in Peru	Empirical	Peru	2014	Logistic regression	*Review of Economics of the Household*	Guerra M.J.M., Mohapatra S., Swallow B.	2019
6	The political economy of women’s entrepreneurship initiatives in Pakistan: reflections on gender, class, and “development”	Theoretical	Pakistan	NA	NA	*Review of International Political Economy*	Roberts A., Mir Zulfiqar G.	2019
7	Women’s empowerment and economic growth: Albany, NY, 1760–1860	Empirical	New York’s Albany County	1760–1860	Descriptive	*Economics Bulletin*	McDevitt C.L., Irwin J.R.	2017
8	Gender equity and the escape from poverty	Theoretical	NA	NA	NA	*Oxford Economic Papers*	Prettner K., Strulik H.	2016
9	Women empowerment and economic development	Literature review	NA	NA	NA	*Journal of Economic Literature*	Duflo E.	2012
10	Gender inequalities and economic growth: a longitudinal evaluation	Empirical	116 countries	1970–1992	Ordinary least square method	*Economic Development and Cultural Change*	Forsythe N., Korzeniewicz R.P., Durrant V.	2000

## Empirical specification

### Pooled OLS and panel model

For empirical estimation, we begin with pooled OLS, panel model using random effect (RE) and maximum likelihood estimator (MLE) procedures, as explained by Mitze et al. (2016), Baltagi (2013), Semykina and Wooldridge (2013) and Allison (2009).^1^ Following this, our empirical panel model takes the following form:

$${y}_{it}=\alpha +{X}_{it}\beta +{u}_{it}$$

where *u*_*it*_ = *μ*_*i*_ + *v*_*it*_, *y* is our dependent variable representing growth, *i* denoting countries and *t* denoting time. *α* is a scalar, *β* is *K* × 1 and *X*_*it*_ is the *i*th observation on *K* explanatory variables. *μ*_*i*_ denotes the unobservable individual-specific effect and *v*_*it*_ denotes the remainder disturbance. In the FE model, the *v*_*it*_ is independent and identically distributed and *X*_*it*_ is assumed independent of the *v*_*it*_ for all *i* and *t*. In the case of FE model, too many parameters are estimated which is a loss of degrees of freedom. Whereas in the random effect model, *μ*_*i*_ is independent of the *v*_*it*_. Additionally, the *X*_*it*_ is assumed independent of the *μ*_*i*_ and *v*_*it*_ for all *i* and *t*. Therefore, the random effect model is an appropriate specification if we are drawing *N* individuals randomly from a large population. Using our variable of interest, Eq. (1) takes the following form:

1 $${grGDP}_{pc, it}=a+\lambda .{logGDP}_{pc,t-1}+\delta {WECON}_{i,t}+\gamma {X}_{i,t}+{\eta}_i+{\tau}_t+{\mu}_{it}$$

where *grGDP*_*pc*, *it*_ is the growth rate of output per capita; *WECON* is women economic rights used as a proxy for women economic rights. *δ* is the coefficient that captures the effect of women economic rights on growth. *X* represents the set of control variables that measures the observable heterogeneity across different countries and *γ* measures the effect of control sets on growth rates. *η* is an unobserved individual effect that is constant over time but varies across countries, *τ* is a time-specific factor constant for all countries and represents the global shocks, e.g. a decline in economic activity or a technology shock, and *μ* is a random error term. *λ* is the coefficient of the lagged value of output per capita (*logy*_*i*, *t* − 1_) used for growth convergence (see Solow, 1956). The detailed definition of all variables is discussed in “Section 4”.

### Spatial econometric models

Moreover, we are also interested in testing the spatial dependence in our analysis of women economic rights. As we know that, many countries are connected geographically and with trade relations. There is the possibility that the level of women economic rights in one location, which we might label *i* (country *i*), depends on the level of women economic rights at location *j* (another country that is located close or far). Formally we might state (for detail see Anselin (1988) and LeSage (2008)):

2 $${y}_i=f\left({y}_j\right),i=1,\dots, n\kern0.75em j\ne i$$

The present study uses spatial autoregressive (SAR) and spatial Durbin model (SDM).^2^

#### SAR specification

The general SAR model can be represented by the following equation:

3 $$Y={\rho}_1{W}_1Y+\gamma X+\mu$$

where *Y* is (*n* × 1) a vector of the dependent variable representing growth rate, *W*_1_ is (*n* × *n*) spatial weight matrices and *ρ*_1_ is a spatial parameter. *X* is (*n* × *k*) the matrix of exogenous variables and *μ* is the traditional disturbance term. *W*_1_*Y* is a spatial lag variable. The *W*_1_ weight matrix (or spatial dependence) captures the spillover effect from neighbouring countries. The reduced form of the extended SAR model along with its likelihood can be shown in the following form:

$$Y-{\rho}_1{W}_1Y= X\gamma +\mu$$

$$u=\left(1-{\rho}_1{W}_1\right)Y- X\gamma$$

4 $$L\left(\gamma, \sigma, {\rho}_1,{\rho}_2\right)={\left(2\pi {\sigma}^2\right)}^{-\frac{n}{2}}\parallel 1-{\rho}_1{W}_1\parallel \exp \left(-\frac{1}{2{\sigma}^2}{\mu}^{\prime}\mu \right)$$

By using our variable of interest, Eq. (3) can be transformed into SAR model, as follows:

5 $${grGDP}_{pc, it}=a+\lambda .{logGDP}_{pc,t-1}+{\rho}_1{W}_1{logGDP}_{pc,t}+\delta {WECON}_{i,t}+\gamma {X}_{i,t}+{\eta}_i+{\tau}_t+{\mu}_{it}$$

In Eq. (5), we transform the specification of Eq. (1) into spatial model (SAR), where we have extra term *ρ*_1_*W*_1_*logGDP*_*pc*, *t* − 1_. The *W*_1_*logGDP* represents the spatial lag of dependent variable, which captures the spillover effect from neighbouring countries growth. *W*_1_ is (*n* × *n*) spatial weight matrices, *ρ*_1_ is a spatial parameter and *λ* is the coefficient of the lagged value of output per capita (*logy*_*i*, *t* − 1_).^3^

#### SDM specification

The spatial Durbin model (SDM) includes a spatial lag of the dependent variable *W*_1_*Y*, as well as the explanatory variable vector *X*, and a spatial lag of the explanatory variable *W*_1_*X*. The SDM model can be expressed by the following equation:

6 $$Y={\rho}_1{W}_1Y+\gamma X+{\gamma}_1{W}_1X+\mu$$

where *W*_1_ is (*n* × *n*) spatial weight matrix; *ρ*_1_ is spatial parameters attached to the spatial lagged dependent variable *W*_1_*Y*. *γ*_1_ is the parameter attached with the spatial lagged explanatory variables *W*_1_*X*, where *γ* usual coefficient is attached with the control variables and *γ*_1_ captures the neighbourhood effect from a set of control variables.

Similar to SAR, the specification of Eq. (6) can be transformed into SDM model, as follows:

7 $${grGDP}_{pc, it}=a+\lambda .{logGDP}_{pc,t-1}+{\rho}_1{W}_1{logGDP}_{pc,t-1}+\delta {WECON}_{i,t}+{\delta}_1{W}_1{WECON}_{i,t}+\gamma {X}_{i,t}+{\gamma}_1{W}_1{X}_{i,t}+{\eta}_i+{\tau}_t+{\mu}_{it}$$

## Data

An unbalance panel dataset, for 171 countries from 1960 to 2016, is used for the analysis. The main variable used is the measure of women’s economic rights that is constructed by Cingranelli and Richards (2010) dataset. This dataset is also known as CIRI Human Rights Database and has been used in many recent studies, see, for example Dreher, Gassebner, and Siemers (2012), Gutmann, Pfaff, and Voigt (2017) and Blanton and Peksen (2016). Concerning the definition of women economic rights, it is very comprehensive and unique which is based on the following internationally recognized definitions rights (Cingranelli and Richards, 2010):

- Equal pay for equal work
- Free choice of profession or employment without the need to obtain a husband or male relative’s consent
- The right to gainful employment without the need to obtain a husband or male relative’s consent
- Equality in hiring and promotion practices
- Job security (maternity leave, unemployment benefits, no arbitrary firing or layoffs, etc.)
- Non-discrimination by employers
- The right to be free from sexual harassment in the workplace
- The right to work at night
- The right to work in occupations classified as dangerous
- The right to work in the military and the police force

The women’s economic rights index, constructed by using the information from the above variables, is used as a proxy for women economic rights that has a value from 0 to 3, where the value 0 indicates no rights, 1 specifies some rights, 2 shows rights under the law while 3 indicate that women’s economic rights were guaranteed by the law and by the government (for detail see Cingranelli & Richards, 2010). A brief summary of women economic rights is presented in Table 2.

**Table 2:** Women economic rights rank and values

0	No rights for women in law and systematic discrimination based on sex may have been built into law.
1	Women had some economic rights under law, but these rights were not effectively enforced.
2	Women had some economic rights under law, and the government effectively enforced these rights in practice while still allowing a low level of discrimination against women in economic matters.
3	All or nearly all of women’s economic rights were guaranteed by law and the government fully and vigorously enforces these laws in practice.

The World Development Indicator (World Bank 2016) dataset is the second source used to collect the data for GDP per capita and other related variables. An overview of the main variables, source and descriptive statistics are presented in Table 3.

**Table 3:** Variables, source and descriptive statistics and data sources

Variables	Source	Obs	Mean	Std. Dev.	Min	Max
Final consumption expenditure (% of GDP)	WDI	7357	82.74877	17.41643	16.27874	341.8554
International trade (% of GDP)	WDI	7822	76.48727	49.00968	0.020999	531.7374
GDP per capita (constant 2010 US$)	WDI	7879	1.653509	6.661927	−185.692	58.61644
Life expectancy at birth, female (years)	WDI	9874	65.32222	12.20505	22.394	86.83
Disappearance (ranking low to high)	CIRI	9677	1.662189	0.648455	0	2
Extrajudicial killing (ranking low to high)	CIRI	9675	1.301499	0.74557	0	2
Political imprisonment (ranking low to high)	CIRI	9682	1.18364	0.832997	0	2
Torture (ranking low to high)	CIRI	9680	0.736777	0.751711	0	2
Freedom of assembly and association (ranking low to high)	CIRI	9685	1.123903	0.837555	0	2
Women economic rights (ranking low to high)	CIRI	9623	1.329523	0.814803	0	3
Political regime (numeric)	CIRI	Country identifier from the Polity Political Regime				
Country identifier (numeric)	CIRI	Country identifier from the United Nations Statistics Division				

Unbalance panel for 171 countries from 1960 to 2016

WDI: World Development Indicators from World Bank

CIRI: available at http://www.humanrightsdata.com/ compiled by Cingranelli and Richards (2010)

## Results

This section presents the empirical results for the impact of women economic rights on growth within a global perspective. As defined earlier, our dependent variable is GDP per capita growth and the main variable of interest is women economic rights (defined as women economic rights) which has a value from 0 to 3. Therefore, the effect of women economic rights is not an absolute effect on growth, but we have one reference group and three treatment groups as follows:

### Reference group: Level 0 (no economic rights for women)

- Treatment groups: women economic rights in three levels
  - Level 1 (specifies some rights)
  - Level 2 (shows rights under the law)
  - Level 3 (shows women’s economic rights were guaranteed by the law and by the government)

Table 4 reports the results for a global sample by using pooled OLS and random effect model by using MLE (maximum likelihood estimator) methods. The effect of women economic rights is the average effect across three different levels of women’s economic rights on GDP per capita growth. The results of Eq. 1 are presented in Table 3 where the first two columns show the OLS results by estimating the model without the panel. The effect of women economic rights is quite large which shows that if a country has women economic rights (average of 3 levels), the growth rate will be 0.55% higher than the country with no women economic rights (level 0). Results reported in columns 3 and 4 employ the panel data model with a random effect model by using the MLE method. In addition, this method control for the other types of individual and country-specific observable characteristics such as human rights variables. Based on the results in column 4, the impact of women economic rights on GDP per capita growth is 0.42% higher than the countries with no women economic rights. Similar findings are reported by recent studies (Dahlum et al., 2022; Kabeer, 2020; Mishra et al., 2020). These results for women economic rights are quite robust to different specifications (for other specifications, see Table 10 in the Appendix).

**Table 4:** Women economic rights and growth (OLS and MLE results for global sample)

Variables	OLS	OLS	MLE	MLE
Women economic rights	0.634***	0.555***	0.595***	0.415***
	0.109	(0.119)	(0.138)	(0.140)
Lag of log GDP percapita	−0.444	−0.873***	−1.262***	−1.392***
	0.0658	(0.0978)	(0.151)	(0.151)
Trade	0.0117***	0.0113***	0.0167***	0.0166***
	0.00148	0.00159	0.0025	0.002
Consumption expenditure	−0.0516	−0.0505***	−0.064***	−0.0551***
	0.0051	0.005	0.007	0.006
Life expectancy		0.0705***	0.070***	0.127***
		0.0104	0.0132	0.0162
Human rights variable				
Disappearance	–	Yes	–	Yes
Extrajudicial killing	–	Yes	–	Yes
Political imprisonment	–	Yes	–	Yes
Torture	–	Yes	–	Yes
Freedom of assembly and association	–	Yes	–	Yes
Regional dummies	–	Yes	–	
Time dummies	No	No	Yes	Yes
Constant	7.842***	6.466***	10.17***	7.388***
	0.779	(0.911)	1.258	(1.511)
Observations		5756		5756
*R*^2^	0.79	0.80	0.77	0.81

Robust standard errors in parentheses by using MLE method of estimation

*, ** and *** represent the level of significance at 1%, 5% and 10%

Panel dimension: time = 1960–2016, countries: 171 in total

Definition of human rights variables is given in the data section

### Subsample (high- and low-income countries)

We also estimate the model for a different group of countries characterized by the level of income. Results from low (low and lower middle income) and high income (high and higher middle income) countries are reported in Table 5. The effect of women economic rights for high-income countries is significant but not for low-income counties. After controlling for different types of observable individual and country-specific heterogeneity, there is 0.45% higher growth for countries with full women economic rights compare to those with zero women economic rights. These results are robust with the findings in Table 2 but not for low-income countries. Low-income countries need particular effort to improve women empowerment as reported by many studies (Dahlum et al., 2022; Kabeer, 2020; Mishra et al., 2020). These results also show that the level of women economic right is not high enough in low- and lower-middle-income countries.

**Table 5:** Women economic rights and growth (OLS and MLE results for high- and low-income countries)

	Low income	High	High	High
Women economic rights	0.204	0.606***	0.570***	0.449**
	(0.239)	0.188	0.171	(0.175)
Lag of log GDP per capita	−1.874***	−1.491***	−2.341***	−2.294***
	(0.289)	−0.233	−0.288	(0.331)
Trade	0.019***	0.0150***	0.0224***	0.0208***
	0.005	0.003	0.003	0.003
Consumption expenditure	−0.075***		−0.0692***	−0.0653***
	0.009		−0.0094	0.009
Life expectancy	0.096***			0.00586
	0.017			0.023
Human rights variable				
Disappearance	Yes	–	–	Yes
Extrajudicial killing	Yes	–	–	Yes
Political imprisonment	Yes	–	–	Yes
Torture	Yes	–	–	Yes
Freedom of assembly and association	Yes	–	–	Yes
Regional dummies	Yes	–	–	Yes
Time dummies	Yes			Yes
Constant	13.68***	13.60***	26.38***	25.28***
	(2.358)	(2.111)	(3.07)	(2.943)
Observations	2896	2860	2860	2860
*R*^2^	0.77	0.79	0.77	0.78

Robust standard errors in parentheses by using MLE method of estimation

*, ** and *** represent the level of significance at 1%, 5% and 10% *p*

Panel dimension: time = 1960–2016, countries: 171 in total

High-income group = 46, low-income group (lower middle + low income) = 88

Definition of human rights variables is given in the data section

### Women economic rights and relative growth

**Fig. 1:**
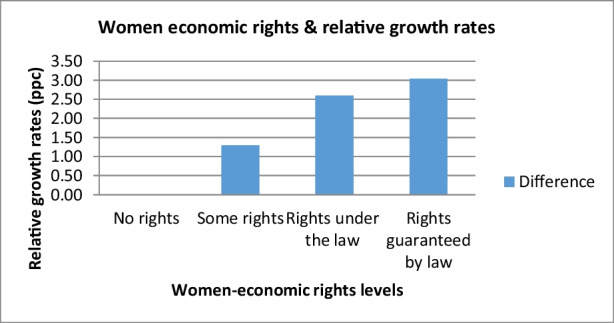
Women economic rights and relative growth rates

As defined earlier that our main variable, women economic rights, is categorized between 0 and 3, where the value 0 indicates no rights, 1 specifies some rights, 2 shows rights under the law while 3 indicate that women’s economic rights were guaranteed by law and by the government (Cingranelli & Richards, 2010). Even within the high-income groups, there is a difference in growth effect. In Table 6, we have estimated the absolute growth and difference in growth rate for the high-income group (based on the estimates from Table 4). There is a significant difference in relative growth rates concerning the different levels of women economic rights. For instance, countries with some economic power have 1.29% higher growth rate than the countries with no economic power. Similarly, countries with economic power under the law have 2.6% higher growth than the treatment group with no power. The countries with full women’s rights have 3% higher growth than the first group. Figure 1 represents the relative growth rates against the different levels of women economic rights.

**Table 6:** Women economic rights and growth accounting (rich countries)

Levels	Women economic power	Absolute	Difference in growth
Level 0	No rights	45.19	–
Level 1	Some rights	46.49	1.29
Level 2	Rights under the law	47.80	2.60
Level 3	Rights guaranteed by law	48.24	3.04

### Spatial analysis results

Before estimating the spatial model, we used a Moran “*I*” test for spatial dependence. The results are reported in Table 7. Based on the Moran test, the null hypothesis of no spatial dependence is rejected which confirms the presence of spatial dependence in the relationship being observed. It means that we must consider the spatial models as an alternative specification to the classical linear regression model otherwise the results will be biased (Anselin & Griffith, 1988; Arbia, 2014; LeSage, 2008). After testing the spatial dependence, the remaining section explains the results of two spatial regression models (SAR and SDM).

**Table 7:** Test results of spatial dependence

	Moran *I*	Statistics	*p* values
GDP per capita	0.194	2.803	0.005
Lag GDP per capita	0.752	10.367	0.000
Trade	0.153	2.42	0.016
Women economic rights	0.454	6.341	0.000

Weigh matrix type: distance-based (binary) based on 3 nearest neighbour

Moreover, the weight matrix is row-standardized

Table 8 reports the results of spatial regression based on the specification in Eqs. (5) and (7) of SAR and SDM models, respectively. Furthermore, we have two panels in Table 5: panel A reports the average results while panel B represents direct, indirect and total effect from women economic rights on growth. In total, we have four regressions with different specifications for comparison. Column (1) and column (2) report the results from SAR and SDM model but without controls, while column (3) and column (4) are more promising specification as it includes both observable (control sets) and unobserved heterogeneity (random effect). Based on panel A, spatial parameter estimate from SAR and SDM model is significant as denoted by “Rho (spatial dependence)” which confirms the existence of spatial dependence.

**Table 8:** Women economic rights and growth (spatial analysis: SAR and SDM models)

Variables	SAR	SDM	SAR	SDM
	(1)	(2)	(3)	(4)
Panel A: average results				
Women Eco-rights	0.428**	0.303	0.296*	0.195
	(0.199)	(0.210)	(0.154)	(0.156)
*W*x [Women Eco-rights]		0.483		0.526**
		(0.313)		(0.237)
Lag of log GDP per capita	−0.275**	−0.400**	−1.490***	−1.551***
	(0.116)	(0.164)	(0.158)	(0.182)
*ρ*_1_ *W*x[Lag of log GDP per capita]		0.0471		0.0452
		(0.191)		(0.241)
Trade	0.00953***	0.00923***	0.000381	0.000737
	(0.00313)	(0.00312)	(0.00265)	(0.00257)
Consumption expenditure			−0.104***	−0.114***
			(0.0112)	(0.0116)
Life expectancy			0.152***	0.133***
			(0.0200)	(0.0284)
Rho (spatial dependence)	0.129***	0.128***	0.216***	0.223***
	(0.0250)	(0.0250)	(0.0231)	(0.0237)
Panel B: direct, indirect and total effects				
Direct effect				
Women Eco-rights	0.433**	0.319	0.301*	0.228
	(0.203)	(0.210)	(0.157)	(0.157)
Indirect effect				
Women Eco-rights	0.0627**	0.567*	0.0782*	0.684**
	(0.0319)	(0.343)	(0.0418)	(0.283)
Total effect				
Women Eco-rights	0.495**	0.886**	0.379*	0.912***
	(0.232)	(0.348)	(0.198)	(0.325)
Direct effect %	87%	36%	80%	25%
Indirect effect %	13%	64%	20%	75%
Observations	2400	2400	2328	2328

Robust standard errors in parentheses by using MLE method of estimation for SAR and SDM models. Control variables for specification (2) and (4) of SDM models: human rights variables (political regime, country identifier, disappearance extrajudicial killing, political imprisonment, torture, freedom of assembly and association), regional dummies, time dummies. Definition of human rights variables is given in the data section. *, ** and *** represent the level of significance at 1%, 5% and 10%. Panel dimension: time = 1991–2014, countries: 100 in total

Based on results from SAR specifications (column 3), the parameter estimate of women economic right is significant with the value of 0.296 and the total effect of women economic rights on GDP growth is also significant with the magnitude of 0.38% (in panel B). It implies that if there is a 1 level increase in women economic rights would result in a 0.379% increase in per capita growth. Out of this around 20% is attributed to the neighbourhood effect from neighbouring countries (indirect or spillover effect) and the remaining 80% comes from each country’s own level of women economic rights (direct impact). It confirms that women’s economic rights is dependent on the level of economic rights in neighbouring countries.

SDM specifications (column 4) include a spatial lag of the dependent variable (*WY*), as well as the explanatory variable vector *X*, and a spatial lag of the explanatory variables (*WX*). The parameter estimate of women economic rights is not significant but the parameter estimate from the spatial lag of women economic rights is significant with the value of 0.526 which confirms the spatial dependence and neighbourhood effect. It implies that the level of women economic rights in a current country is positively affected by the level of women economic rights by its neighbouring countries. Moreover, in SDM specification, we can see that neighbourhood (indirect) effect is 75% while the country’s own effect from women economic rights is only 25%. It further highlights the importance of spatial spillover from neighbouring countries. The additional implication of our findings is that collaborative efforts by the neighbouring countries regarding women economic rights can benefit every country in the regions. Our results support the efforts by European Union regarding women’s economic rights in the region (Cichowski, 2004; Ellina, 2004; Dahlum et al., 2022; Kabeer, 2020; Mishra et al., 2020).

#### Spatial analysis on subsample (high- and low-income groups)

We also estimate the model for a different group of countries characterized by the level of income. Results from low (low and lower middle income) and high income (high and higher middle income) countries are reported in Table 9.^4^ The effect of women economic rights for high-income countries is significant, but not for low-income counties. Table 9 reports the direct, indirect and total effects of women economic rights on growth. Concerning high-income countries, the direct effect from the SAR model is greater than the indirect (spillover) effect while in the SDM model, the indirect (spillover) effect is greater than the direct effect. The results are also consistent with Table 5 about SDM specification and in general supported by the previous studies (Dahlum et al., 2022; Kabeer, 2020; Mishra et al., 2020). Insignificant results for developing countries could be due to the heterogeneous impact of women economic rights. In addition, further investigation can be carried out on the subsample of the group of low-income countries. The group can be divided into different geographical regions and/or ethnic backgrounds.

**Table 9:** Women economic rights and growth (SAR and SDM models for high-income group)

Variables	High**-**income countries		Low-income countries	
	SAR	SDM	SAR	SDM
Direct,indirect and total effects:				
Direct effect				
Women Eco-rights	0.406***	0.373***	0.00256	0.0143
	(0.148)	(0.143)	(0.308)	(0.305)
Indirect effect				
Women Eco-rights	0.255**	0.640*	0.00147	−0.0496
	(0.0994)	(0.342)	(0.0426)	(0.599)
Total effect				
Women Eco-rights	0.662***	1.013***	0.00404	−0.0353
	(0.244)	(0.389)	(0.349)	(0.696)
Direct effect %	61%	37%	63%	40%
Indirect effect %	39%	63%	37%	60%
Observations	1200	1200	1128	1128

Robust standard errors in parentheses by using MLE method of estimation for SAR and SDM models. Control variables for specification (2) and (4) of SDM models: human rights variables (political regime, country identifier, disappearance extrajudicial killing, political imprisonment, torture, freedom of assembly and association), regional dummies, time dummies. Definition of human rights variables is given in the data section. *, ** and *** represent the level of significance at 1%, 5% and 10%. Panel dimension: time = 1991–2014, countries: 100 in total

## Summary and conclusion

This study tries to explore the nexus between women economic rights and economic growth. Particularly, it tests the hypothesis that women economic rights leads to economic growth. However, economic growth in many countries not only depends upon their own level of women’s economic rights but also on the level of women economic rights in the neighbouring countries through foreign direct investment, international trade, social and cultural linkages and globalization (Elson, 1999; Neumayer & De Soysa, 2011; Wichterich, 2000). Therefore, to correctly identify the impact of women’s economic rights, this study employs spatial econometric tools to correctly identify the impact of women economic rights from their own and neighbouring countries. For empirical analysis, we used panel data over the period 1960 to 2016 for a global sample of 171 countries. For estimation purposes, we employ OLS, MLE with robust standard error, as well as the spatial models (SAR and SDM).

The results of this study show that women’s economic rights significantly increase the economic growth for the global sample. This relationship is significant regardless if we use estimation techniques (pooled OLS and MLE) or the one that takes care of spatial dependence (SAR and SDM). This result suggests that improving women’s economic rights is an important step in achieving sustainable development goals. However, this study also confirms the relative growth rates differs with respect to different levels of women economic rights.

Concerning the different groups of countries, we find that higher-income countries are benefiting more from women’s economic rights compared to low-income countries. The reason could be that the level of women’s economic rights in developed countries to some extent is more than the developing counties. There is a need to provide more work opportunities in developing world, which will assist in achieving sustainable development.

From spatial analysis, we find that significant spatial dependence exists among the group of countries in our sample. Particularly, in the SDM model, we find that the neighbourhood (indirect) effect is 75% while a country’s own effect from women economic rights is only 25%. It confirms that spatial spillover from neighbouring countries is significant which implies that collaborative efforts by the neighbouring countries regarding women economic rights can benefit every country in the regions. The results about different economies, characterized by income, are robust where the effect of women economic rights is stronger in developed countries than in developing countries. Although the results related to low-income countries are not as significant as expected, however, the general findings confirm that providing more economic rights not only improves the economic development in that country but also has similar impact on its neighbouring countries. This confirms the spillover effect from neighbourhood and that is the unique result of this study.

Our results support the efforts of the European Union regarding women economic rights in the region (Cichowski, 2004; Ellina, 2004).

From a policy perspective, this study provides crucial empirical evidence that enhancing women’s economic rights can strengthen not only growth but also provide a path towards sustainable development. Especially, after the recent pandemic (COVID-19), special attention should be provided to underdeveloped countries for creating more work opportunities for women. This should be the priority areas and can be considered as a crucial determinant of economic development. Therefore, focussing on women’s empowerment through enhancing their economic rights should be considered as appropriate growth strategy in the new millennia. Further, policies related to educating women can enhance their empowerment that leads to further economic development.

Besides strong empirical evidence, this study has few limitations to carryout comprehensive research. For instance, the CIRI data is unique and have many important elements related to gender; however, the time period is limited which is an important constraint of this study. For future research, it would be interesting to investigate the effectiveness of women’s economic rights at disaggregate levels and by using more updated dataset. For example, the analysis could be extended for choosing subsample of countries, especially dividing the sample with regard to different regions (ASEAN, GCC or ASEAN sample) or with countries with different levels of women empowerment or micro-level study for a smaller sample.

## Appendix

**Table 10:** Women economic rights and growth (SAR and SDM models for high- and low-income groups)

Variables	High-income countries		Low-income countries	
	SAR	SDM	SAR	SDM
	(1)	(2)	(3)	(4)
Panel A:average results				
Women Eco-rights	0.396***	0.336**	0.0151	0.0281
	(0.144)	(0.146)	(0.319)	(0.315)
*W*x[Women Eco-rights]		0.259		−0.0258
		(0.222)		(0.583)
Lag of log GDP per capita	−1.285***	−1.526***	−1.915***	−2.187***
	(0.206)	(0.229)	(0.459)	(0.494)
*ρ*_1_ *W*x[Lag of log GDP per capita]		0.0452		0.0452
		(0.241)		(0.241)
Trade	0.0067***	0.0072***	0.00403	0.00530
	(0.00224)	(0.00221)	(0.00677)	(0.00671)
Consumption expenditure	−0.0197	−0.0222	−0.154***	−0.162***
	(0.0129)	(0.0144)	(0.0194)	(0.0208)
Life expectancy	0.0718**	0.0905**	0.165***	0.121***
	(0.0311)	(0.0388)	(0.0322)	(0.0391)
Rho (spatial dependence)	0.411***	0.413***	0.116***	0.111***
	(0.0291)	(0.0292)	(0.0347)	(0.0366)
Panel B:direct,indirect and total effects				
Direct effect				
Women Eco-rights	0.406***	0.373***	0.00256	0.0143
	(0.148)	(0.143)	(0.308)	(0.305)
Indirect effect				
Women Eco-rights	0.255**	0.640*	0.00147	−0.0496
	(0.0994)	(0.342)	(0.0426)	(0.599)
Total effect				
Women Eco-rights	0.662***	1.013***	0.00404	−0.0353
	(0.244)	(0.389)	(0.349)	(0.696)
Direct effect %	61%	37%	63%	40%
Indirect effect %	39%	63%	37%	60%
Observations	1200	1200	1128	1128

Robust standard errors in parentheses by using MLE method of estimation for SAR and SDM models. Control variables for specification (2) and (4) of SDM models: human rights variables (political regime, country identifier, disappearance extrajudicial killing, political imprisonment, torture, freedom of assembly and association), regional dummies, time dummies. Definition of human rights variables is given in the data section. *, ** and *** represent the level of significance at 1%, 5% and 10%. Panel dimension: time = 1991–2014, countries: 100 in total

## Footnotes

1 The RE is preferable to the FE (fixed effect) because it avoids the loss of degrees of freedom caused by too many parameters if the error term is assumed to be random (Baltagi, 2013).

2 For detail about the basic SAR and SDM model see LeSage (1998) and Anselin and Griffith (1988).

3 The detailed definition of all variables is discussed in “Section 4”.

4 Full estimation results for high- and low-income countries can be found in the Appendix Table 10.
